# Supplementary material for: A combined mathematical and experimental approach reveals the drivers of time-of-day drug sensitivity in human cells
Source: Commun Biol. 2025 Mar 25;8:491. doi: 10.1038/s42003-025-07931-1 (PMC11937577; doi:10.1038/s42003-025-07931-1)
Supplement: Supplementary file 1 — Supplementary Information [file 42003_2025_7931_MOESM1_ESM.pdf]

Supplementary Figures

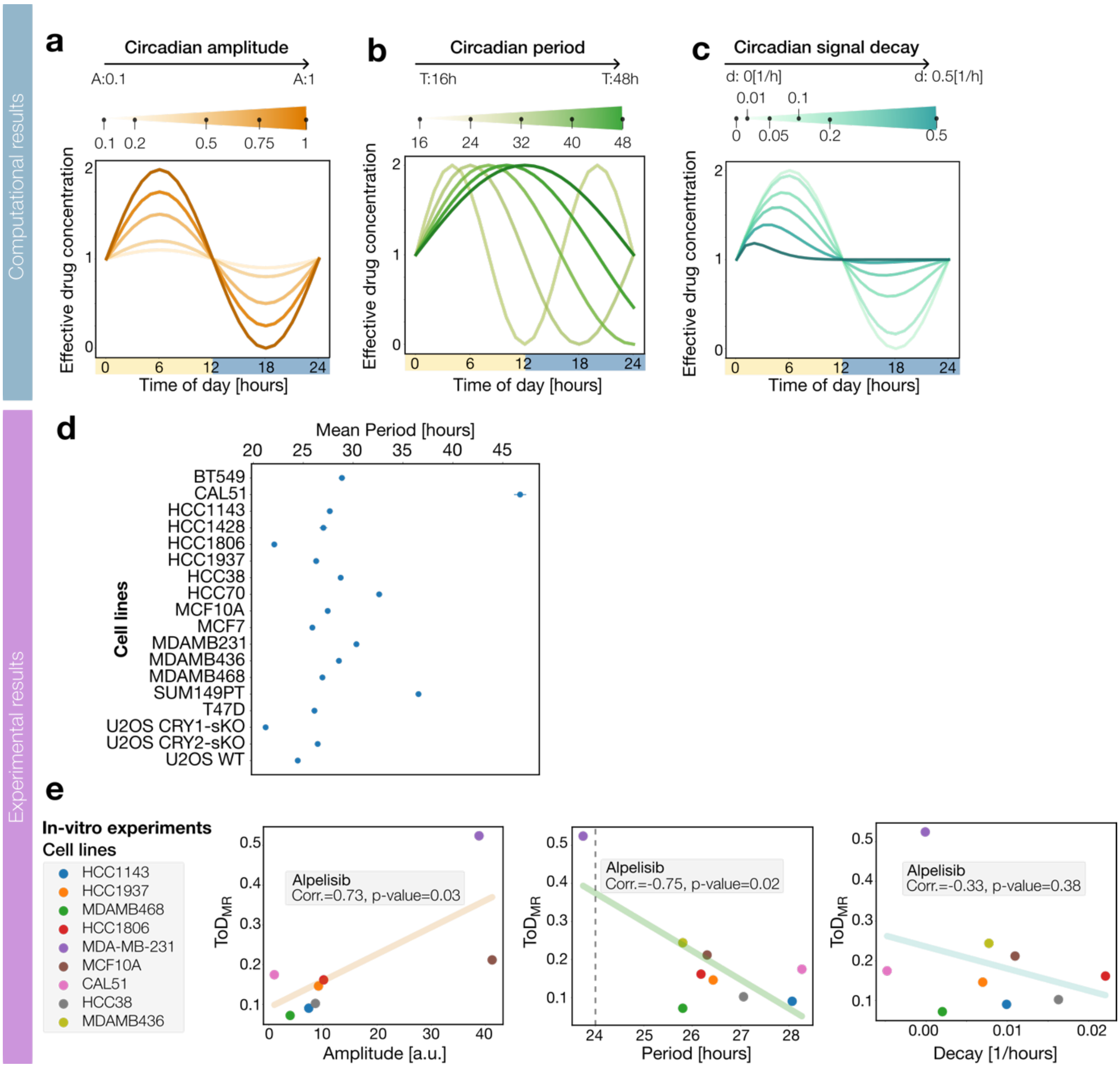

Figure S1. Circadian clock properties shape time-of-day drug responses

a, Simulation of the effective drug concentration with circadian clock modulation for varying amplitude values.

b, Simulation of the effective drug concentration with circadian clock modulation for varying period values.

c, Simulation of the effective drug concentration with circadian clock modulation for varying signal decay values.

d, Mean period of the circadian rhythm obtained from the fitting of bioluminescence recordings of different tumor cell lines.

e, Maximum range of the experimental ToD response curve of Alpelisib for the amplitude (left), period (middle), and signal decay (right) values of different cell lines (see the legend on the left). The legend inside the plot shows the correlation coefficient with the corresponding p-value.

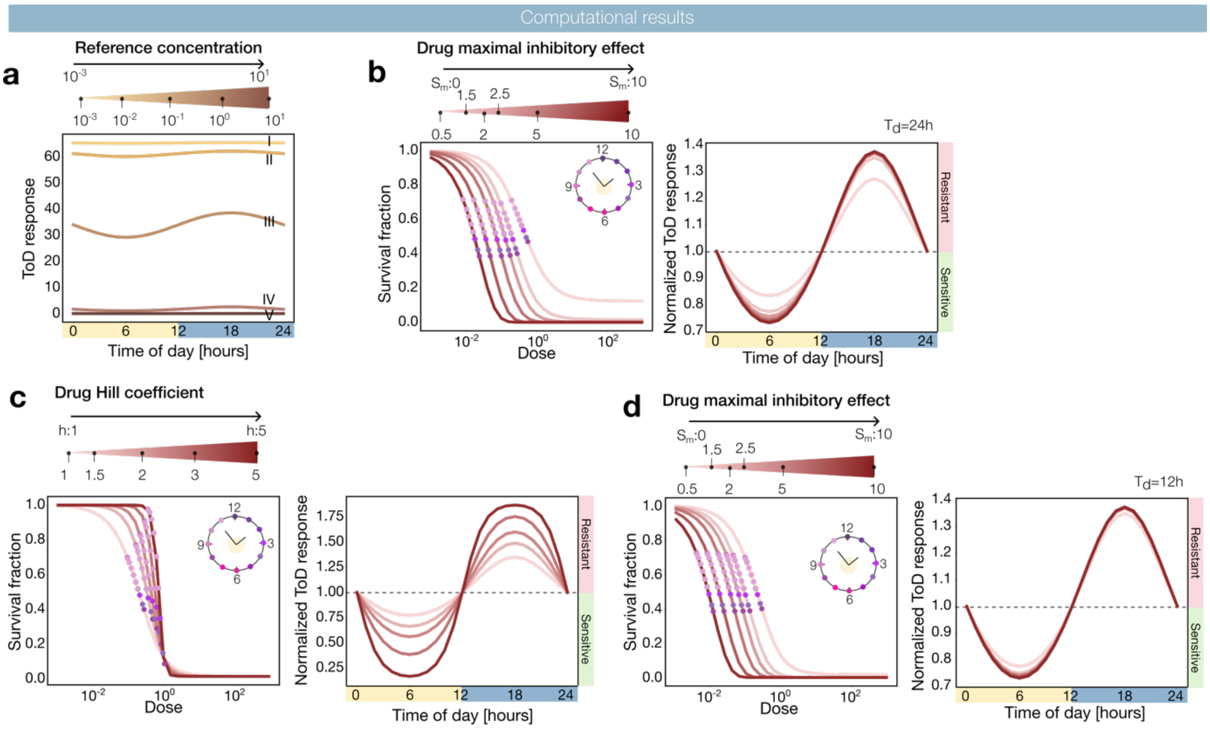

Figure S2. Cytostatic drug parameters affect time-of-day drug response

a, ToD response curve without normalization for different reference dose concentrations corresponding to Figure 3c.

b, Left: simulated survival curve for different values of the drug maximal inhibitory effect S_m, corresponding to a cytostatic drug with a doubling time of 24 hours. The color gradient of the curves shows different S_m values from the range [0, 10]. The dots on the survival curve represent drug administration at different times within a day. Right: simulated ToD response curve for the different S_m values with the same doubling time.

c, Left: simulated survival curve for different values of the Hill coefficient for a cytostatic drug. The color gradient shows different Hill coefficient values within [1, 5]. The dots on the survival curve represent drug administration at different times within a day. Right: simulated ToD response curve for the different Hill coefficient values for a doubling time of 24 hours.

d, Left: simulated survival curve for different values of the drug maximal inhibitory effect S_m, corresponding to a cytostatic drug with a doubling time of 12 hours. Right: simulated ToD response curve for the different S_m values with a division rate of 12 hours.

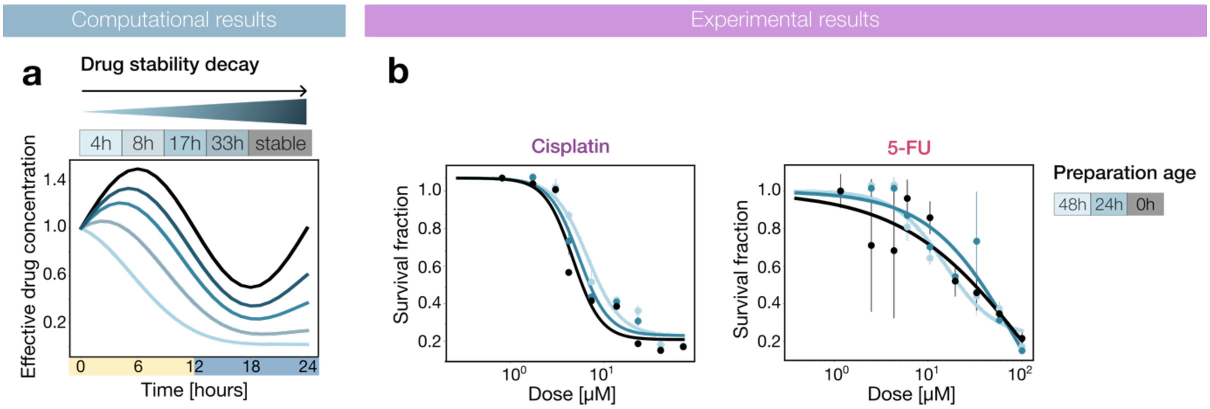

Figure S3. Drug stability affects time-of-day drug responses

a, Simulation of the effective drug concentration with circadian clock modulation for different half-life values of the drug.

b, Experimental survival fraction corresponding to Cisplatin (left) and 5-FU (right) for different preparation ages: 0, 24, and 48 hours. The points represent the mean value and the error bars the standard deviation derived from three technical replicates.

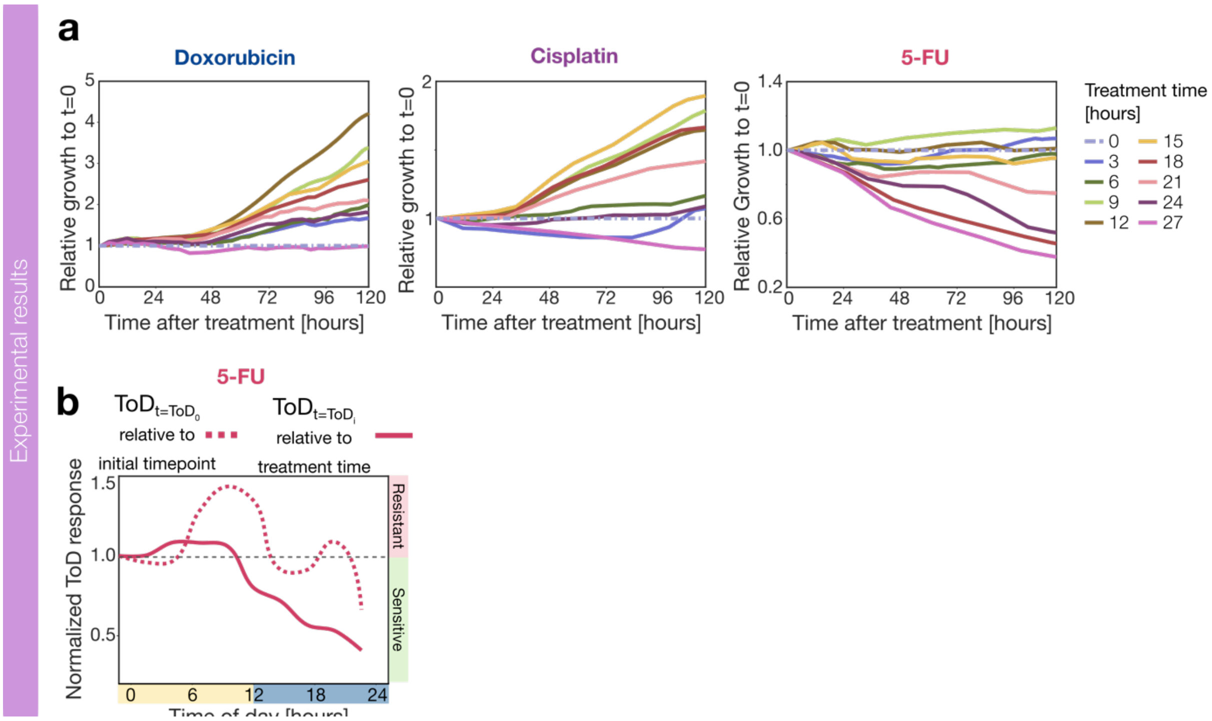

Figure S4. Cell normalization to initial or treatment time changes the drug response

a, Experimental relative growth to the first time point for Doxorubicin, Cisplatin, and 5-FU for different treatment times.

b, Experimental ToD response curves for 5-FU relative to the initial (dashed line) or treatment (continuous line) time points.

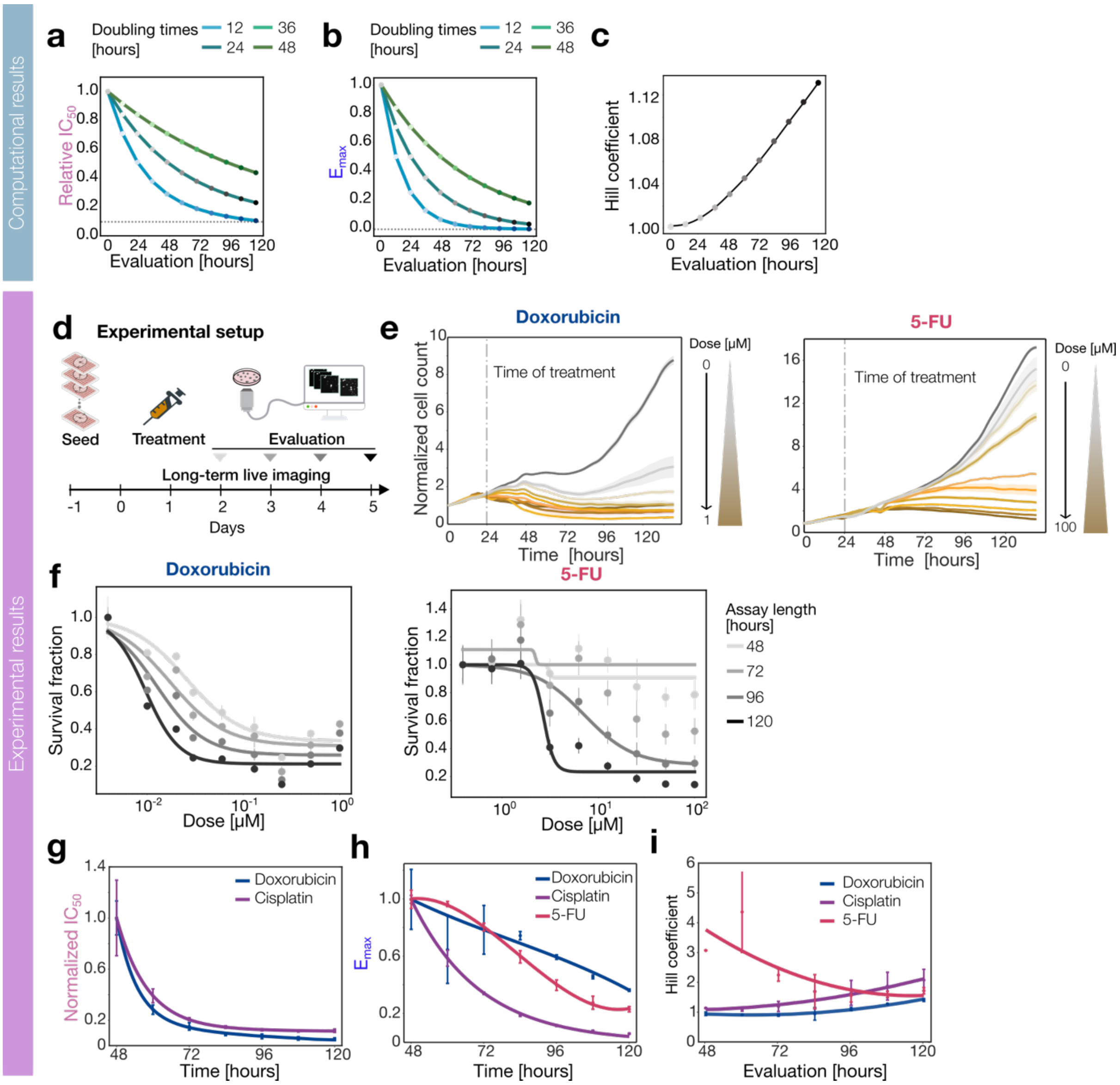

Figure S5. Assay length changes the time-of-day response

a, Relative half-response concentration IC_{50} for different doubling times and assay lengths from 0 to 120 hours obtained from the fitting of Figure 5g.

b, Maximal drug effect E_{max} for different doubling times and assay lengths from 0 to 120 hours obtained from the fitting of Figure 5g.

c, Hill coefficient for different doubling times and assay lengths from 0 to 120 hours obtained from the fitting of Figure 5g.

d, Experimental setup to study the effect of the time of evaluation on the Time-of-Day response curve.

e, Normalized cell counts to the first time point for different doses (grey to brown color gradient) for Doxorubicin (left) and 5-FU (right).

f, Experimental survival curve for different assay lengths from 48 to 120 hours (grey to black gradient color) for Doxorubicin and 5-FU. The points represent the mean value and the error bars the standard deviation derived from three technical replicates.

g, Experimental relative IC_{50} of Doxorubicin and Cisplatin for different assay lengths from 48 to 120 hours obtained from the fitting of Figure 5k. The points represent the mean value, and the error bars represent standard errors of the residuals from the curve fit.

h, Experimental E_{max} of Doxorubicin, Cisplatin and 5-FU for different assay lengths obtained from the fitting of Figure 5k. The points represent the mean value, and the error bars represent standard errors of the residuals from the curve fit.

i, Experimental Hill coefficient for Doxorubicin, Cisplatin and 5-FU for different assay lengths obtained from the fitting of Figure 5k. The points represent the mean value, and the error bars represent standard errors of the residuals from the curve fit.

Supplementary Tables

Table S1. Fitting of circadian signals from the bioluminescence recordings performed in [1]:

Cell lines	Amplitude	Error_A	Period	Error_T	decay	Error_d
BT549_Bmal1	34,3059	0,7822	31,2747	0,4825	0,0696	0,0021
BT549_Per2	73,0440	2,5115	26,9595	0,1890	0,0386	0,0018
CAL51_Bmal1_Luc	20,7445	0,4810	63,4313	0,9714	0,0555	0,0012
CAL51_Per2_Luc	34,8408	1,1885	21,6948	0,0723	0,0175	0,0009
HCC1143_Bmal1_Luc	20,4684	0,3733	28,9896	0,0543	0,0163	0,0004
HCC1143_Per2_Luc	42,3118	1,6211	26,3378	0,1096	0,0192	0,0010
HCC1428_Bmal1	104,3347	3,8819	24,5657	0,5299	0,0744	0,0042
HCC1428_Per2_1De	282,9361	5,3475	29,4709	0,2080	0,0432	0,0012
HCC1806_Bmal1_Luc	43,7509	1,0472	22,5086	0,1537	0,0479	0,0017
HCC1806_Per2_Luc	608,7309	17,6141	21,6866	0,1062	0,0372	0,0015
HCC1937_Bmal1_Luc	45,1181	0,7253	26,5190	0,0782	0,0387	0,0008
HCC1937_Per2_Luc	44,8460	1,3753	26,0378	0,0862	0,0156	0,0008
HCC38_Bmal1_Luc	52,5534	1,0106	26,0834	0,1572	0,0657	0,0015
HCC38_Per2_Luc	236,7617	7,2806	32,0932	0,2381	0,0403	0,0015
HCC70_Bmal1	39,4558	0,5888	29,3204	0,0849	0,0300	0,0006
HCC70_Bmal1_Luc	22,7162	0,2086	36,4376	0,1682	0,0628	0,0007
HCC70_Per2	242,4247	4,1006	30,9801	0,0834	0,0197	0,0005
MCF10A_Bmal1	199,6399	1,7549	27,8485	0,0434	0,0245	0,0003
MCF10A_Bmal1_1Dex	211,6253	2,1641	27,7414	0,0468	0,0228	0,0003
MCF10A_Per2	15,7360	0,3719	26,9844	0,0690	0,0160	0,0006
MCF10A_Per2_1Dex	20,3720	0,4808	27,2025	0,0714	0,0160	0,0006
MCF7_Bmal1_Luc	290,3761	7,2458	25,7394	0,0431	0,0058	0,0004
MCF7_Bmal1_Luc_1uMDe	1860,3104	19,5383	24,7865	0,0290	0,0194	0,0003
MCF7_Per2	128,2281	1,5006	26,3971	0,0370	0,0168	0,0003
MCF7_Per2_Luc	139,9322	1,5997	26,1594	0,0364	0,0174	0,0003
MCF7_Per2_Luc_1uMDe	122,1063	1,6716	26,2644	0,0366	0,0141	0,0003
MDAMB231_Bmal1	429,4149	7,4844	25,0125	0,1509	0,0532	0,0013
MDAMB231_Per2	290,9343	14,2545	46,2872	0,3680	0,0181	0,0012
MDAMB436_Per2	277,7907	9,3060	28,5765	0,1494	0,0259	0,0012
MDAMB468_Bmal1_Luc	6,0412	0,1828	27,0968	0,0521	0,0048	0,0004
MDAMB468_Per2	180,0086	3,8109	26,5769	0,0359	0,0067	0,0003
SUM149PT_Bmal1_Luc	72,5485	1,0764	40,5378	0,2733	0,0471	0,0009
SUM149PT_Per2_Luc	757,4817	15,5573	32,5699	0,2457	0,0480	0,0014
T47D_Bmal1	114,9224	2,3294	25,0264	0,0911	0,0314	0,0009
T47D_Per2	84,5190	1,5044	27,7751	0,0696	0,0217	0,0006
U2OS CRY1-sKO_BLH	300,2585	13,2461	21,2311	0,1055	0,0231	0,0014
U2OS CRY2-sKO_BLH	749,0248	13,7055	26,4510	0,0330	0,0059	0,0003
U2OS WT_BLH	955,7247	31,9482	24,9269	0,0523	0,0081	0,0005
U2OS WT_Per2_Luc	257,2436	4,9592	23,5046	0,0197	0,0000	0,0002

References

1. Ector, C. *et al.* Time-of-day effects of cancer drugs revealed by high-throughput deep phenotyping. 2023.11.30.569380 Preprint at https://doi.org/10.1101/2023.11.30.569380 (2024).
