# Supplementary material for: A combined mathematical and experimental approach reveals the drivers of time-of-day drug sensitivity in human cells
Source: Commun Biol. 2025 Mar 25;8:491. doi: 10.1038/s42003-025-07931-1 (PMC11937577; doi:10.1038/s42003-025-07931-1)
Supplement: Supplementary file 2 — Reporting summary [file 42003_2025_7931_MOESM2_ESM.pdf]

nature portfolio

Corresponding author(s): Adrian E. Granada

Last updated by author(s): Feb 19, 2025

Reporting Summary

Nature Portfolio wishes to improve the reproducibility of the work that we publish. This form provides structure for consistency and transparency in reporting. For further information on Nature Portfolio policies, see our Editorial Policies and the Editorial Policy Checklist.

Statistics

For all statistical analyses, confirm that the following items are present in the figure legend, table legend, main text, or Methods section.

n/a	Confirmed	
☐	☒	The exact sample size (*n*) for each experimental group/condition, given as a discrete number and unit of measurement
☐	☒	A statement on whether measurements were taken from distinct samples or whether the same sample was measured repeatedly
☒	☐	The statistical test(s) used AND whether they are one- or two-sided<br>*Only common tests should be described solely by name; describe more complex techniques in the Methods section.*
☒	☐	A description of all covariates tested
☒	☐	A description of any assumptions or corrections, such as tests of normality and adjustment for multiple comparisons
☐	☒	A full description of the statistical parameters including central tendency (e.g. means) or other basic estimates (e.g. regression coefficient) AND variation (e.g. standard deviation) or associated estimates of uncertainty (e.g. confidence intervals)
☒	☐	For null hypothesis testing, the test statistic (e.g. *F*, *t*, *r*) with confidence intervals, effect sizes, degrees of freedom and *P* value noted<br>*Give P values as exact values whenever suitable.*
☒	☐	For Bayesian analysis, information on the choice of priors and Markov chain Monte Carlo settings
☒	☐	For hierarchical and complex designs, identification of the appropriate level for tests and full reporting of outcomes
☒	☐	Estimates of effect sizes (e.g. Cohen's *d*, Pearson's *r*), indicating how they were calculated

Our web collection on statistics for biologists contains articles on many of the points above.

Software and code

Policy information about availability of computer code

Data collection	Incucyte analysis software (Sartorius, v2022A), for acquisition and image analysis of population live-cell imaging data.<br>Lumicycle analysis software (Actimetrics), for luciferase signal acquisition (https://actimetrics.com/products/lumicycle/).<br>The experimental raw data and data tables generated in this study have been deposited in the Figshare database under the identifier: https://doi.org/10.6084/m9.figshare.28423709
Data analysis	The implemented code used for this study can be found here: https://github.com/Granada-Lab/drivers_ToD_drug_sensitivity

For manuscripts utilizing custom algorithms or software that are central to the research but not yet described in published literature, software must be made available to editors and reviewers. We strongly encourage code deposition in a community repository (e.g. GitHub). See the Nature Portfolio guidelines for submitting code & software for further information.

Data

Policy information about availability of data

All manuscripts must include a data availability statement. This statement should provide the following information, where applicable:

- Accession codes, unique identifiers, or web links for publicly available datasets
- A description of any restrictions on data availability
- For clinical datasets or third party data, please ensure that the statement adheres to our policy

All the data generated and used for this investigation is available here: https://doi.org/10.6084/m9.figshare.28423709.

Research involving human participants, their data, or biological material

Policy information about studies with human participants or human data. See also policy information about sex, gender (identity/presentation), and sexual orientation and race, ethnicity and racism.

Reporting on sex and gender	n/a
Reporting on race, ethnicity, or other socially relevant groupings	n/a
Population characteristics	n/a
Recruitment	n/a
Ethics oversight	n/a

Note that full information on the approval of the study protocol must also be provided in the manuscript.

Field-specific reporting

Please select the one below that is the best fit for your research. If you are not sure, read the appropriate sections before making your selection.

☒ Life sciences ☐ Behavioural & social sciences ☐ Ecological, evolutionary & environmental sciences

For a reference copy of the document with all sections, see nature.com/documents/nr-reporting-summary-flat.pdf

Life sciences study design

All studies must disclose on these points even when the disclosure is negative.

Sample size	Sample size was not pre-determined. According to the standards in the field, two independent bioluminescence experiments were conducted with two to three technical replicates. For live-cell imaging, we analyzed a minimum of four images for each condition, sourced from three independent plates.
Data exclusions	No data was excluded from the analysis.
Replication	Bioluminescence experiments were conducted twice to confirm their reproducibility.<br>Live-imaging experiments were carried out a single time because of the comprehensive data generated in three separate plates. The evaluation of each condition was based on a detailed analysis of the captured individual images.
Randomization	No randomization was applied as the study did not involve large cohorts for treatment and control groups.
Blinding	Investigators were not blinded to group allocation during either data collection or analysis. Given the nature of the data and the analytical methods used, blinding was not deemed relevant to the integrity or objectivity of the study results.

Reporting for specific materials, systems and methods

We require information from authors about some types of materials, experimental systems and methods used in many studies. Here, indicate whether each material, system or method listed is relevant to your study. If you are not sure if a list item applies to your research, read the appropriate section before selecting a response.

Materials & experimental systems

n/a	Involved in the study
☒	☐ Antibodies
☐	☒ Eukaryotic cell lines
☒	☐ Palaeontology and archaeology
☒	☐ Animals and other organisms
☒	☐ Clinical data
☒	☐ Dual use research of concern
☒	☐ Plants

Methods

n/a	Involved in the study
☒	☐ ChIP-seq
☒	☐ Flow cytometry
☒	☐ MRI-based neuroimaging

Eukaryotic cell lines

Policy information about cell lines and Sex and Gender in Research

Cell line source(s)	HCC1143, HCC1806, HCC1937, HCC38, and MDAMB468 cells were obtained from the American Type Culture Collection. BT549, CAL51, MDAMB231, MDAMB436, and SUM149PT cells were kindly provided by the Sorger lab (Harvard Medical School, Ludwig Cancer Center, Boston, USA). MCF10A and MCF7 cells were kindly gifted by the Brugge lab (Harvard Medical School, Ludwig Cancer Center, Boston, USA). GIMEN and SH-SY5Y cells were provided by the Schulte lab (Universitätsklinikum Tübingen, Clinic for Pediatrics and Adolescent Medicine, Thübingen, Germany) and the U2OS reporter cell lines by the Kramer lab (Charité, Institute for Medical Immunology, Berlin, Germany).
Authentication	The cells were authenticated using SNP-typing technology, Multiplexion GmbH.
Mycoplasma contamination	Cell lines were tested for mycoplasma contamination and showed no infection, nor any signs of health decline.
Commonly misidentified lines (See ICLAC register)	No commonly misidentified cell line was used in this study.

Plants

Seed stocks	n/a
Novel plant genotypes	n/a
Authentication	n/a
